# Dissimilar Ligands Bind in a Similar Fashion: A Guide to Ligand Binding-Mode Prediction with Application to CELPP Studies

**DOI:** 10.3390/ijms222212320

**Published:** 2021-11-15

**Authors:** Xianjin Xu, Xiaoqin Zou

**Affiliations:** 1 Dalton Cardiovascular Research Center, University of Missouri, Columbia, MO 65211, USA; xuxian@missouri.edu; 2 Department of Physics and Astronomy, University of Missouri, Columbia, MO 65211, USA; 3 Department of Biochemistry, University of Missouri, Columbia, MO 65211, USA; 4 Institute for Data Science and Informatics, University of Missouri, Columbia, MO 65211, USA

**Keywords:** molecular similarity principle, dissimilar ligand, binding-mode prediction, protein–ligand interactions, drug discovery, CELPP

## Abstract

The molecular similarity principle has achieved great successes in the field of drug design/discovery. Existing studies have focused on similar ligands, while the behaviors of dissimilar ligands remain unknown. In this study, we developed an intercomparison strategy in order to compare the binding modes of ligands with different molecular structures. A systematic analysis of a newly constructed protein–ligand complex structure dataset showed that ligands with similar structures tended to share a similar binding mode, which is consistent with the Molecular Similarity Principle. More importantly, the results revealed that dissimilar ligands can also bind in a similar fashion. This finding may open another avenue for drug discovery. Furthermore, a template-guiding method was introduced for predicting protein–ligand complex structures. With the use of dissimilar ligands as templates, our method significantly outperformed the traditional molecular docking methods. The newly developed template-guiding method was further applied to recent CELPP studies.

## 1. Introduction

Molecular similarity (or chemical similarity) is one of the most important concepts in cheminformatics and plays a pivotal role in the drug development process, such as in the optimization of lead compounds for drug products [1,2,3,4,5]. The molecular similarity principle states that molecules with similar structures are likely to have similar properties. In protein–ligand interaction studies, molecules that are structurally similar tend to bind in a similar fashion, and therefore induce similar bioactivities [6,7]. Meanwhile, it is also common that a small variation in the molecular structure can dramatically change the binding behavior of the ligand in a protein binding pocket, and therefore result in distinct bioactivities [6,7].

Many computational methods have been developed for molecular similarity measurement. These methods can be categorized into three classes: 1D similarity, 2D similarity, and 3D similarity. In 1D chemical similarity search methods, molecular properties, such as molecular weight and polar surface area, are normally used as the descriptors for similarity calculations. 2D similarity methods use 2D descriptors of molecules, such as substructure-based molecular fingerprints. For example, the Tanimoto coefficient of fingerprints is one of the commonly used 2D chemical similarity calculation methods [8]. In 3D similarity methods, the information derived from 3D coordination is used for similarity measurements. An example of 3D methods is SHAFTS, which uses molecular shape and pharmacophore features for 3D similarity calculation [9,10]. In this study, we analyzed the relationship between the molecular similarities of small molecules and their binding modes in proteins. The 3D molecular similarity, which can characterize ligand conformations, was used as one of the major metrics in this study (see Materials and Methods). Detailed reviews regarding molecular similarity calculations are referred to in the References [5,8,9]. It is worth mentioning that the existing molecular similarity calculation methods are designed to find similar molecules and exclude dissimilar molecules for a query ligand. 

However, we do not have much knowledge about the relationship among dissimilar ligands, which are much more common than similar ligands, as any molecule has many more dissimilar forms than similar forms. This is due to two main reasons. First, molecules that are structurally dissimilar tend to bind in a dissimilar fashion, and therefore induce different bioactivities. This assumption makes the comparison of the binding fashions of dissimilar molecules less attractive. Despite this being true for many cases, dissimilar ligands can also result in similar bioactivities [6,11]. Second, it is challenging to compare the binding modes of two ligands with distinct structures, and therefore such a strategy is at large and urgently needed.

In this study, we introduced an intercomparison strategy to compare the binding modes of ligands with different molecular structures. Then, we constructed a structural dataset consisting of 2,619 protein–ligand complex structures and 17 different proteins and applied the intercomparison approach to this dataset. Moreover, we developed a novel template-guiding method for ligand binding-mode prediction. We applied the method to a large-scale dataset from Continuous Evaluation of Ligand Pose Prediction (CELPP) that was released by the Drug Design Data Resource (D3R) [12,13,14,15,16].

## 2. Results

### 2.1. Comparison of the Ligand Binding Modes

#### 2.1.1. Ligand Root-Mean-Square Deviation (RMSD) vs. Molecular Similarity

**Figure 1:**
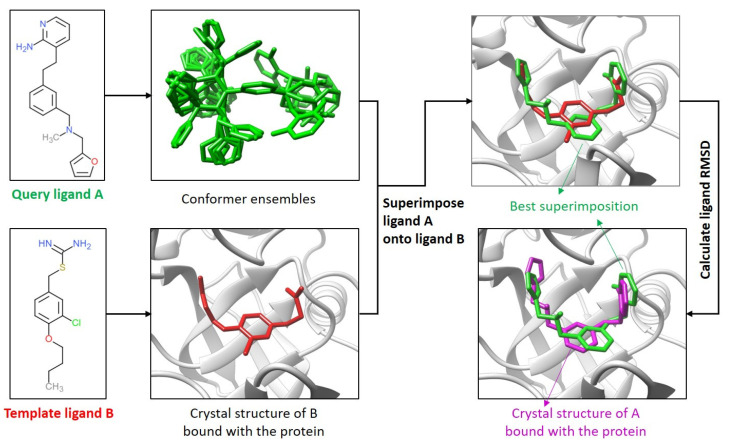
A flowchart for intercomparison of the binding modes of two structurally dissimilar ligands.

**Figure 2:**
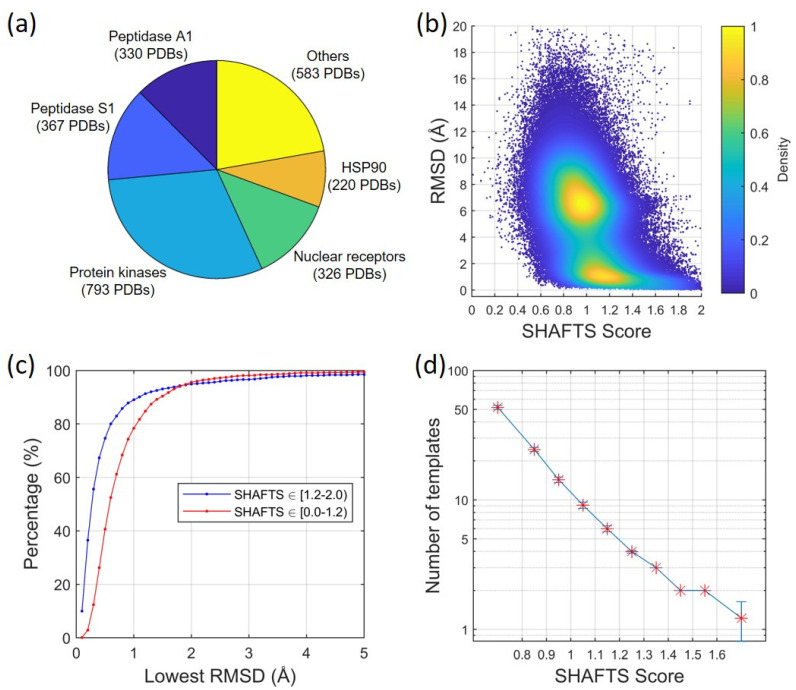
Relationship between ligand binding modes (RMSD) and ligand 3D similarities (SHAFT Scores). (**a**) A newly constructed structural dataset consisting of 17 different proteins and 2,619 protein–ligand complexes, which is listed in Table S1. (**b**) The distribution of the ligand RMSDs vs. the corresponding SHAFTS scores for all the cases in the protein–ligand dataset. (**c**) The percentages of the cases in which at least one template ligand (structurally similar or dissimilar) was successfully found with the RMSD lower than a threshold (referred to as the lowest RMSD). (**d**) The relationship between the SHAFTS score and the minimum number of template ligands among which at least one good template ligand can be found. A good template ligand is the one based on which a query ligand can be predicted with a low RMSD binding mode (≤2.0 Å).

Using our intercomparison approach (Figure 1, also see Section 4.1), we analyzed the binding modes of different ligands on their target proteins in our newly constructed dataset. The dataset consists of 17 different proteins with a total of 2619 protein–ligand complex structures, as shown in Figure 2a and Table S1. These proteins were selected because they have multiple crystal structures that are co-bound with different ligands.

For each protein in the dataset, each ligand was compared with all other ligands using molecular 3D similarities (i.e., the SHAFTS score) and the corresponding RMSDs. Specifically, a ligand in one crystal structure was used as a query ligand, and the ligands in the remaining crystal structures were used as the template ligands. If there were N crystal structures (corresponding to N different ligands) for a protein, then each ligand was compared with (N-1) ligands in the remaining crystal structures, yielding a total of N×(N−1) SHAFTS score–RMSD pairs.

Figure 2b plots the distribution of ligand RMSDs vs. the corresponding SHAFTS scores for all 17 proteins in the dataset. Not surprisingly, the cases with higher similarity scores tended to have lower RMSD values, which is consistent with the Molecular Similarity Principle. The correlation R between the RMSDs and the SHAFTS scores is −0.54. Using the default cutoff of 1.2 for the SHAFTS score, 19.0% of the cases fall into similar regions (SHAFTS score ≥ 1.2). 64.3% of them achieved low RMSD values (≤2.0 Å), which represent similar binding modes. For the cases with a similarity score of no less than 1.6, the percentage of low RMSD cases increased to 93.9%.

On the other spectrum of the SHAFTS scores, the dissimilar ligands (i.e., SHAFTS score < 1.2) make up 81.0% of the total cases, among which the percentages of dissimilar and similar binding modes are 85.1% and 14.9%, respectively. Interestingly, in addition to a densely populated region that was centered around the SHAFTS score of 1.0 and the RMSD of 6.0 Å, another dense region was found at the low RMSD region that was centered around the SHAFTS score of 1.1 and the RMSD of 1.0 Å, showing that dissimilar ligands can bind in a similar fashion.

Furthermore, the SHAFTS score consists of two components, the ShapeScore (molecular shape similarity) and the FeatureScore (pharmacophore feature similarity). Both ShapeScore and FeatureScore range from 0 to 1, in which 0 represents no similarity and 1 corresponds to an identical shape or identical pharmacophore feature. Figure S2a,b show the distribution of ligand RMSDs in our protein–ligand dataset based on the ShapeScores and FeatureScores, respectively. Like those found in Figure 2b using the combined score (i.e., the SHAFTS score), the cases with higher similarity scores (i.e., ShapeScores or FeatureScores) tended to have lower RMSD values, which is consistent with the Molecular Similarity Principle. The correlation R between the RMSDs and the ShapeScores and FeatureScores is −0.52 and −0.46, respectively, indicating that low RMSD values can also have low ShapeScores or low FeatureScores, which is the basis of this study.

To further investigate the importance of the two different scores, ShapeScore and FeatureScore, we calculated the percentages of the cases with low RMSD values (≤2.0 Å) for different ranges of the two scores. The bin size was set to 0.1 for both scores. The results for different combinations of the two scores are shown in Figure S2c. The value “0” in a cell means there were not enough data for the calculations (i.e., fewer than 100 cases). Not surprisingly, the cases with both a high ShapeScore and a high FeatureScore have a much higher chance to achieve low RMSD values, whereas the cases with both low ShapeScore and low FeatureScore tended to have high RMSD values. For the cases with a high ShapeScore (0.7–0.9) but a low FeatureScore (0.1–0.3), the percentages of the cases with low RMSD values range from about 21–33%, indicating that the molecular shape plays an important role in protein–ligand binding. However, the molecular shape alone is not sufficient to determine the ligand binding mode in a protein pocket. Other features, such as pharmacophore, are also important to ligand binding.

In addition to the ligand RMSD distributions based on 3D molecular similarities (such as SHAFTS scores), Figure S3 shows the results based on 2D fingerprint molecular similarities, i.e., the Tanimoto coefficient. Like the results based on 3D similarities, the cases with high Tanimoto coefficients tended to have low RMSD values (R = −0.27). In addition to a densely populated region around the Tanimoto coefficient of 0.4 and the RMSD of 6.0 Å, another densely populated region was found at the low RMSD region, centered around the Tanimoto coefficient of 0.55 and the RMSD of 1.0 Å, showing that dissimilar ligands can bind in a similar fashion.

#### 2.1.2. Ligand RMSD vs. Number/Quality of Templates

Figure 2c shows the percentages of the cases in which at least one template ligand (structurally either similar or dissimilar) were successfully found with an RMSD lower than the threshold. The lowest RMSD value was considered for each query ligand. For similar ligands (i.e., SHAFTS score ≥ 1.2), 94.9% (89.0%) of query ligands had at least one template ligand with the RMSD no larger than 2.0 Å (1.0 Å). Surprisingly, high percentages of the low RMSD cases were also observed for the dissimilar ligands (i.e., SHAFTS score < 1.2). Specifically, 95.6% (78.4%) of the query ligands had at least one template ligand with RMSD no greater than 2.0 Å (1.0 Å). The results suggest that it is not rare for dissimilar ligands to bind in a similar fashion in protein–ligand binding.

Figure 2d shows the relationship between the SHAFTS score and the minimum number of template ligands among which at least one good template ligand can be found. Here, a good template ligand is defined as the template based on which a query ligand can achieve a low RMSD value (≤2.0 Å), as explained in the Supplementary Materials. The results show that the minimum number of template ligands decreases with an increase in the SHAFTS score. For the SHAFTS score range of [1.2, 1.3), at least four template ligands were required in order to include a good template ligand for a query ligand. This number reduced to two when the SHAFTS scores were greater than 1.4. For the dissimilar ligands, significantly more template ligands were required in order to include a good template ligand for a query ligand. For the SHAFTS score range of [1.1, 1.2), the minimum number of template ligands was six. This number was as high as 52 when the SHAFTS scores were lower than 0.8.

### 2.2. Ligand Binding-Mode Prediction

The above analysis revealed that similar ligands tended to have a similar binding mode, which is consistent with the Molecular Similarity Principle. Although dissimilar ligands tended to bind in dissimilar fashions, we could always find at least one template ligand with a similar binding mode for a query ligand. However, the molecular similarity score alone was unable to distinguish good template ligands from co-bound ligands of the protein for a query ligand, especially when only dissimilar template ligands were available, as shown in the section of “Ligand RSMD vs. molecular similarity” in Supplementary Materials. This phenomenon is not surprising, because molecular similarity considers only ligand information and ignores the contributions from binding protein partners.

**Figure 3:**
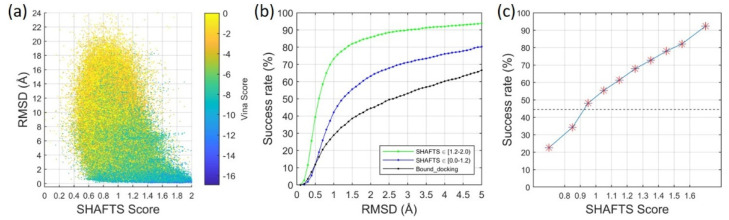
The performance of the template-guiding method on binding-mode prediction. (**a**) Binding scores of the best superimposed query ligands on the corresponding proteins after local refinements. (**b**) Success rates of binding-mode prediction for the template-guiding method when similar template ligands (SHAFTS ≥ 1.2) or dissimilar template ligands (SHAFTS < 1.2) were used. The performance of molecular docking using bound protein structures was also presented for reference. Different RMSD values were used as the respective thresholds for success rate calculations. (**c**) Success rates for the cases using the template ligands with different levels of qualities (characterized by SHAFTS scores). The RMSD value of 2.0 Å was set as the threshold. The broken line corresponds to the success rate of bound docking.

To address this issue, we employed a scoring function for molecular docking in order to evaluate the interactions between a superimposed query ligand and a target protein. Briefly, for a query ligand and a template ligand, the best superimposed conformer of the query ligand and the co-bound protein structure of the template ligand was used as the initial structure for a local refinement process in the molecular docking program, AutoDock Vina [17] (See Materials and Methods for details). The Vina score was calculated with the refined binding mode. This process was performed for all of the binding modes that are shown in Figure 2b, and the results after the refinements are shown in Figure 3a. Remarkably, models with low RMSD values tended to have more negative (i.e., better) Vina scores (protein–ligand binding scores) and were not correlated with molecular similarity scores (SHAFTS scores). A detailed analysis of Figure 3a showed that when using similar ligands as the templates (SHAFTS score ≥ 1.2), the Vina score was able to identify a good template ligand (corresponding ligand RMSD ≤ 2.0 Å) as the top candidate (the lowest Vina binding score) for 79.5% of the cases. Remarkably, even when using dissimilar ligands as the templates (SHAFTS score < 1.2), the Vina score was able to rank a good template ligand as the top candidate for 56.7% of the cases.

Figure 3b shows the total success rates of binding-mode predictions that are based on a hybrid scoring function, which combines both the Vina score that characterizes the protein–ligand interaction and the SHAFTS score that features the molecular similarity (See Materials and Methods for details). For each query ligand, the binding-mode prediction was defined as a success if the ligand RMSD of the top predicted mode was less than the threshold. Different RMSD thresholds were used for the success rate calculations. The bound docking was also calculated as a reference, as molecular docking methods usually achieve the best performance with bound docking [15,16]. AutoDock Vina was employed for bound docking and only the Vina score was used for ranking models. In bound docking, the protein structure was extracted from the experimentally determined complex structure whereas the ligand structure was generated from the SMILES string. In the example of an RMSD threshold of 2.0 Å, the success rate was 85.8% when using similar ligands as the templates, and 63.2% when using dissimilar ligands as the templates. Both success rates were significantly higher than the performance of bound docking (44.5%).

Figure 3c shows the success rates of the binding-mode predictions for the cases using template ligands with different similarity qualities (measured by the SHAFTS scores). In this figure, an RMSD value of 2.0 Å was used as the threshold. For the cases with the SHAFTS scores ranging between 0.8 and 1.6, the bin size was set to 0.1, and the success rate for each bin (represented by the red stars) was calculated based on the cases with the SHAFTS scores in each bin size. The success rates of the cases with the SHAFTS scores below 0.8 or greater than 1.6 were also calculated and plotted in the figure. As expected, the success rate increased with the SHAFTS score, which marked the molecular similarity between a query ligand and a template ligand. Inspiringly, the use of even low-quality template ligands (i.e., the SHAFTS scores between 0.9 and 1.0) with our template-guiding method achieved a success rate (48.2%) that was higher than bound docking (44.5%, represented by the broken line in the figure).

### 2.3. CELPP Dataset

**Figure 4:**
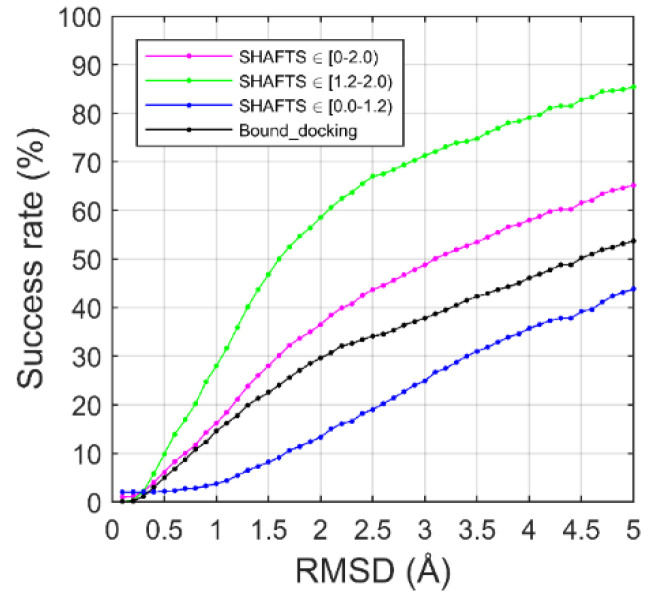
Binding-mode prediction on the CELPP targets using the template-guiding method and a bound docking method.

We applied the template-guiding method for ligand binding-mode prediction to recent CELPP targets. The results are shown in Figure 4. As a reference, bound dockings were also performed for the CELPP targets. In bound docking, the protein structure was extracted from the experimentally determined complex structure. The ligand 3D structure was generated from the SMILES string and then flexibly docked to the binding pocket on the protein using AutoDock Vina. Here, only the top predicted model of each target was used for the success rate calculations. Encouragingly, our newly developed method outperformed bound docking. In the example of an RMSD threshold of 2.0 Å, the template-guiding method achieved a success rate of 36.5%, vs. 29.6% for bound docking.

We further analyzed the performances of our template-guiding method when different qualities of templates were used in the prediction. As shown in Figure 4, the template-guiding method yielded dramatically better performance than bound docking when high-quality templates (SHAFTS score ≥ 1.2) were used. However, bound docking achieved better performance than the template-guiding method when only the low-quality templates (SHAFTS score < 1.2) were used. Using an RMSD value of 2.0 Å as the threshold, the success rates of the template-guiding method using the high-quality templates and the low-quality templates were 58.5% and 13.3%, respectively. It is worth mentioning that the performance of our template-guiding method depends on the number of the templates that are used in the prediction, particularly when only low-quality templates are available (Figure 2d). For example, when the SHAFTS scores of the template ligands and the query ligand were between 1.1 and 1.2, the template-guiding method required about six different template ligands to achieve a correct binding-mode prediction. The number of required template ligands increased with the decline of the SHAFTS score. This number increased to 52 when the SHAFTS scores were lower than 0.8. In the CELPP studies, there were few template ligands available for some targets. For example, there were fewer than four template ligands available for about 24% of the CELPP targets in which only low-quality templates were available. Thus, the low success rate of the template-guiding method using low-quality templates for the CELPP targets were mainly due to an insufficient number of available templates.

## 3. Discussion

In the intercomparison strategy for comparing the binding modes of two different ligands, it is noteworthy that the RMSD value changes when the two ligands are switched, i.e., the RMSD from using ligand B as the query ligand and ligand A as the template is different from the RMSD of the other way around. For example, in Figure 1, the RMSD of ligand B that was superimposed with the co-bound ligand A was 1.36 Å, which was slightly different from the RMSD of ligand A when it was superimposed with the co-bound ligand B (1.42 Å). Based on the protein–ligand complex structure dataset that we built, the correlation (R) between the two RMSDs was 0.71. The correlation increased to 0.9 for the low RMSD values (≤ 2.0 Å), as shown in Figure S1. In some cases, the RMSD of ligand A that was superimposed with the co-bound ligand B may have been significantly different from the RMSD of ligand B that was superimposed with ligand A. This difference was mainly due to the different sizes of the query and template ligands, as the RMSD was calculated based on the query ligand. The difference was also due to the slightly different superimposition when the query ligand and the template ligand were switched.

In this study, we find out that a surprising number of dissimilar ligands can bind in a similar fashion on a target protein. However, the mechanism behind this phenomenon is unclear. A possible mechanism could be hidden in the conformational plasticity of protein binding pockets, which can bind with dissimilar ligands. In this study, ligand flexibility was fully considered, but proteins were treated as rigid. In the future we will study the mechanism of this important phenomenon and the challenge of protein flexibility. 

## 4. Materials and Methods

### 4.1. Comparison of the Ligand Binding Modes

The existing methods of the binding-mode comparison of two ligands in a protein pocket are restricted to identical ligands. Here, we developed an intercomparison strategy for the binding-mode comparison of different ligands, as explained in Figure 1. One ligand (A) works as a query ligand, and the other ligand (B) as a template ligand. Two human β-secretase 1 inhibitors, ligand A (PDB ID: 3kn0) and ligand B (PDB ID: 3kmx) [18], were used as an example. The protein structures in these two PDB entries were matched using the MatchMaker tool of UCSF Chimera [19].

First, up to 200 three-dimensional (3D) conformers were generated from a SMILES string for the query ligand A, using the OMEGA2 program (Version 2.5.1.4, OpenEye Scientific Software, Santa Fe, NM, USA. http://www.eyesopen.com, accessed on 10 April 2021) [20,21].

Next, each conformer of ligand A was superimposed with the co-bound structure of the template ligand B (PDB ID: 3kmx), using the SHAFTS program [9,10]. SHAFTS is a method for 3D similarity calculation and superimposition that considers both the overlay of molecular shapes (ShapeScore) and the matching of pharmacophore features (FeatureScore). The SHAFTS score ranges from 0 to 2, with 0 representing no similarity and 2 corresponding to the identical ligand. A value of 1.2 is the default cutoff in SHAFTS for the distinction of similar and dissimilar ligands.

Then, the superimposed conformers of the query ligand A were ranked by their SHAFTS scores based on the template ligand B. The conformer of ligand A with the highest SHAFTS score was used for binding-mode evaluation. In the case shown in Figure 1, the highest SHAFTS score of the two ligands was 1.1, indicating they were dissimilar ligands.

Finally, the root-mean-square deviation (RMSD) was calculated for the binding mode of ligand A that was superimposed with the template ligand B (i.e., the conformer that had the highest similarity score) and the co-bound structure of ligand A in the crystal structure (PDB ID: 3kn0), which was 1.42 Å. It is worth emphasizing that the RMSD was calculated between the binding modes of the same molecule (ligand A). The commonly used cutoff, 2.0 Å, for the distinction of similar and dissimilar binding modes in protein–ligand studies was employed in this study. Therefore, the query ligand A shared a similar binding mode with the template ligand B in this case, despite the fact that the two ligands had dissimilar structures.

### 4.2. Minimum Number of Template Ligands vs. SHAFTS Score

To find out the minimum number of template ligands among which there was at least one good template ligand (see the data shown in Figure 2d), we performed the following calculations. For the cases in a range of SHAFTS scores (see Figure 2b), n models were randomly selected, among which the minimum RMSD value was kept. This process was repeated 500 times. The average value and the standard error of the 500 minimum RMSDs were calculated. The value of n was initialized to 1, and was increased until the average value of the minimum RMSDs plus the standard error was not higher than 2.0 Å. The maximum value of n corresponded to the minimum number (*N_min_*) of the template ligands that contained at least one good template. Then, the whole procedure was repeated 100 times. The average value and the standard error of the 100 values of *N_min_* were calculated and shown in Figure 2d.

The above calculation was performed for the cases in different ranges of SHAFTS scores. The bin size was set to 0.1 for the cases with SHAFTS scores between 0.8 and 1.6. The cases with SHAFTS scores less than 0.8 or higher than 1.6 were calculated separately.

### 4.3. Construction of a Structural Dataset of Protein–Ligand Complex Structures

To systematically compare the binding modes of dissimilar ligands, we constructed a diverse database of protein–ligand complex structures. Specifically, crystal or NMR structures containing protein and co-bound ligands were downloaded from Protein Data Bank (PDB, as of 1 October 2018) [22]. A PDB structure was discarded if: (1) the ligand was covalently linked to the protein; (2) there were multiple ligands in the same binding pocket (i.e., containing cofactors); (3) the ligand interacted with multiple proteins; (4) the number of heavy atoms of the ligand was less than 7; or (5) the molecular weight of the ligand was less than 140 Da or larger than 800 Da.

To remove the entries in which only a few contacts were present between a ligand and its protein partner, we calculated the percentage of the buried surface area of a ligand upon binding. Specifically, we calculated the solvent-accessible surface area (SASA) of the ligand alone (by deleting the protein partner from the complex structure), which is referred to as SASAlig, using the program Naccess V2.1.1 [23]. We also calculated the SASA of the ligand in the protein–ligand complex, which is referred to as SASAlig’. Thus, the buried percentage of the ligand equals ΔSASA/SASAlig, where ΔSASA = SASAlig—SASAlig’. The entry was discarded if the value of the buried percentage was below 50%.

Then, the PDB structures were grouped according to the protein UniProt IDs. If multiple binding sites were found for a target protein, the structures containing ligands in the major binding site (i.e., the binding site with the largest number of co-bound ligands) were kept. If there was more than one PDB structure that contained the same ligand for the same protein, the structure with the lowest R-free value was kept.

As a result, a total of 17 protein targets were selected, as listed in Table S1 of the Supplementary Materials. The number of the co-bound ligands for each target ranges from 77 to 330. For each protein target, all of the complex structures were matched using the “MatchMaker” tool in UCSF Chimera [19] (Version 1.10,) based on the protein structures. 

### 4.4. Template-Guiding Method for Ligand Binding-Mode Prediction

The template-guiding method consists of three steps: superimposition, local refinement, and re-scoring. The three steps are described in detail as follows.

Step 1: Superimposition. The intercomparison strategy was used in this step. Briefly, for a query ligand, up to 200 3D conformers were generated from a SMILES string using the OMEGA2 program [20,21]. Then, these conformers were superimposed with the co-bound structure of the template ligand using the SHAFTS program [9,10]. The superimposed conformer with the highest SHAFTS score in complex with the target protein was used as the input for the subsequent local refinement step.

Step 2: Local refinement. Superimposition of the query ligand with the template ligands often results in atomic clashes with the protein structure. Therefore, a local refinement step was carried out with AutoDock Vina [17], using the best superimposed query ligand together with the protein structure of the template ligand as the starting structure. The protein was treated as a rigid body, and the ligand was fully flexible. For the local refinement, the option “local_only” was turned on, and other parameters were set as the default.

Step 3: Re-scoring. The refined models were re-ranked using a hybrid scoring function, which combined the protein–ligand binding score (i.e., the Vina score) and the 3D molecular similarity between the query ligand and the template ligand (i.e., the SHAFTS score). The hybrid score was calculated as follows:

Hybrid_Score = Vina_score + α ∗ SHAFTS_score (1)

here, α is a constant for a target protein. It was determined by the best docking scores (i.e., the most negative Vina score) of all the ligands on the target protein. Specifically, α = min (Vina scores)/2. Therefore, the binding score and the ligand similarity score have similar contributions to the final score because the SHAFTS score is no more than 2.

In addition to the above template-guiding method, bound docking was also performed and used for reference. In bound docking, the protein structure was extracted from the experimentally determined complex structure whereas the ligand structure was generated from the SMILES string. In this study, AutoDock Vina was employed for bound docking and the Vina score was used for ranking the models generated by AutoDock Vina.

For a query ligand, a binding-mode prediction was defined to be a success if the ligand RMSD of the top predicted mode was less than the threshold (default value: 2.0 Å). Then, the success rate of a prediction strategy was the percentage of success among all of the query ligands in the dataset. 

### 4.5. CELPP Dataset

To promote the improvement of the existing methods and the development of new approaches for predicting protein–ligand interactions, the Drug Design Data Resource (D3R, starting from 2015) continues to release valuable benchmarking datasets containing experimentally determined binding structures and affinity data [12,13,14,15]. Recently, the D3R Team has developed the Continuous Evaluation of Ligand Pose Prediction (CELPP) [16,24], which is an automated workflow to process and evaluate the challenge of protein–ligand binding-mode prediction. CELPP is held weekly, in which the targets are prepared based on pre-released data from the Protein Data Bank (PDB), including the ligands and the sequence of their target proteins.

In this study, we analyzed the prediction results of our template-guiding method based on 2617 targets that were submitted from week 10 of 2019 to week 45 of 2020. A total of 3298 targets were released during these 85 weeks. Failed submissions were mainly due to two reasons: (1) template structures were not available, or (2) query ligands contained uncommon atoms. In addition, targets were discarded if query ligands were docked to the wrong binding sites, in which the distance between the geometry centers of a predicted binding site and of a real binding site (i.e., the binding site in the released experimental complex structure) was larger than 10 Å. The RMSD calculations failed for some cases in which the experimentally determined structures had missing ligand atoms. Finally, a total of 1,766 targets were analyzed in this study. 

### 4.6. Calculation of Ligand RMSDs

The RMSD was used to assess the quality of a predicted binding mode with respect to the mode in the corresponding experimental complex structure. Specifically, the protein structures were matched using the MatchMaker tool of UCSF Chimera [19], and the RMSDs of the heavy atoms in the ligands were calculated using the maximum common substructure (MCS) functionality of the OEChem Python toolkit (version 2.5.1.4, OpenEye Scientific Software, Santa Fe, NM, USA. http://www.eyesopen.com, accessed on 10 April 2021) [20,21]. The MCS functionality enables ligand atom renumbering and takes account of compound symmetries that are often observed in ligand superimposition.

## 5. Conclusions

In this study, we analyzed the binding modes of ligands with different molecular structures using a new intercomparison strategy. The results revealed that a surprising number of very dissimilar ligands can bind in a similar fashion, based on which we developed a new template-guided method for predicting protein–ligand complex structures. With the use of dissimilar ligands as templates, our method significantly outperformed traditional molecular docking methods.

## Data Availability

Our constructed dataset of protein–ligand complex structures is openly available through the link http://zougrouptoolkit.missouri.edu/materials/pro_lig_mode_analysis.tar.gz (accessed on 10 April 2021). Predicted binding modes of CELPP targets are available through the link http://zougrouptoolkit.missouri.edu/materials/CELPP/CELPP_week10_2019_week45_2020.tar.gz (accessed on 10 April 2021).

## Supplementary Materials

The following are available online at https://www.mdpi.com/article/10.3390/ijms222212320/s1.
