# Supplementary material for: Dissimilar Ligands Bind in a Similar Fashion: A Guide to Ligand Binding-Mode Prediction with Application to CELPP Studies
Source: Int J Mol Sci. 2021 Nov 15;22(22):12320. doi: 10.3390/ijms222212320 (PMC8625032; doi:10.3390/ijms222212320)
Supplement: Supplementary file 1 [file ijms-22-12320-s001.zip › ijms-1425845-supplementary.pdf]

Supplementary Materials for

Dissimilar Ligands Bind in a Similar Fashion: A Guide to Binding Mode Prediction with Application to CELPP Studies

Xianjin Xu, Xiaoqin Zou*

Dalton Cardiovascular Research Center, Department of Physics and Astronomy, Department of Biochemistry, Informatics Institute, University of Missouri, Columbia, MO 65211, USA

*Correspondence to: Xiaoqin Zou, Email: zoux@missouri.edu

Table S1 The constructed dataset of the protein-ligand complex structures.

Protein family/UniProt ID	Protein name	Number of PDBs
Peptidase A1		
BACE1_HUMAN	Beta-secretase 1	330
Peptidase S1		
THRB_HUMAN	Prothrombin	141
TRY1_BOVIN	Cationic trypsin	118
FA10_HUMAN	Coagulation factor X	108
Protein kinase		
CDK2_HUMAN	Cyclin-dependent kinase 2	296
MK14_HUMAN	Mitogen-activated protein kinase 14	165
PIM1_HUMAN	Serine/threonine-protein kinase pim-1	128
CHK1_HUMAN	Serine/threonine-protein kinase Chk1	121
AURKA_HUMAN	Aurora kinase A	83
Heat shock protein 90		
HS90A_HUMAN	Heat shock protein HSP 90-α	220
Nuclear hormone receptor		
ESR1_HUMAN	Estrogen receptor	210
PPARG_HUMAN	Peroxisome proliferator-activated	116
Others		
BRD4_HUMAN	Bromodomain-containing protein 4	205
TNKS2_HUMAN	Tankyrase-2	120
HYES_HUMAN	Bifunctional epoxide hydrolase 2	93
PK3CG_HUMAN	PI3-kinase subunit gamma	88
POL_HV1B1	Gag-Pol polyprotein	77
Total	17 different proteins	2619

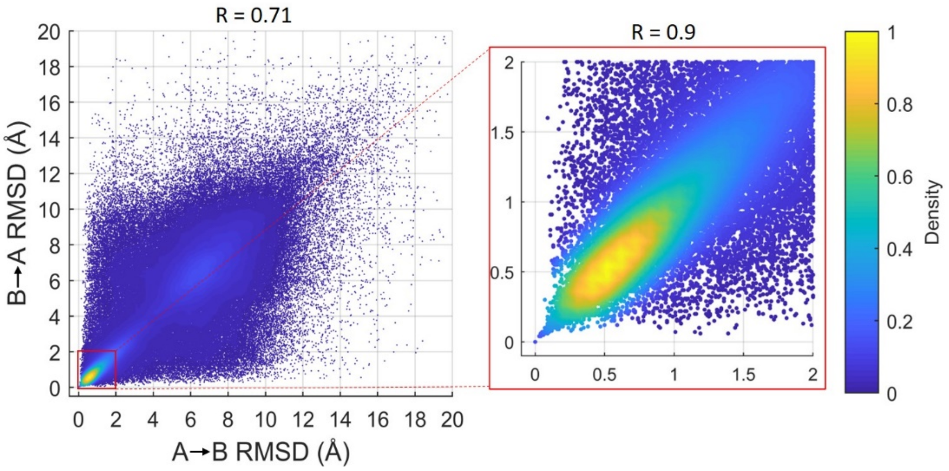

Figure S1 Relationship between the calculated RMSDs of two superimposed ligands when the query ligand and the template ligand are switched. RMSD of A→B is the RMSD between ligand A and ligand B when A is set as the query ligand and B as the template ligand. RMSD of B→A is vice versa. The right panel shows the cases that have low RMSD values (≤ 2.0 Å).

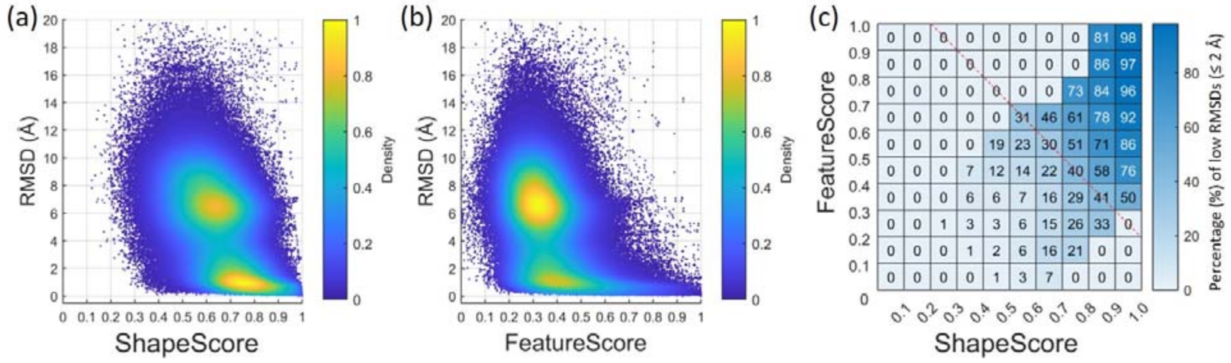

Figure S2 Elaborative studies of Figure 2(b). Distributions of the ligand RMSDs *vs* corresponding ShapeScores (a) or FeatureScores (b) for all the cases in the protein-ligand dataset. (c) Percentages of the cases with low RMSD values (≤ 2.0 Å) for different ranges of ShapeScore and FeatureScore. The value "0" in a cell means there were not enough data (fewer than 100 cases) for the calculation. The broken red line corresponds to the cases with the SHAFTS score (namely, ShapeScore + FeatureScore) of around 1.2.

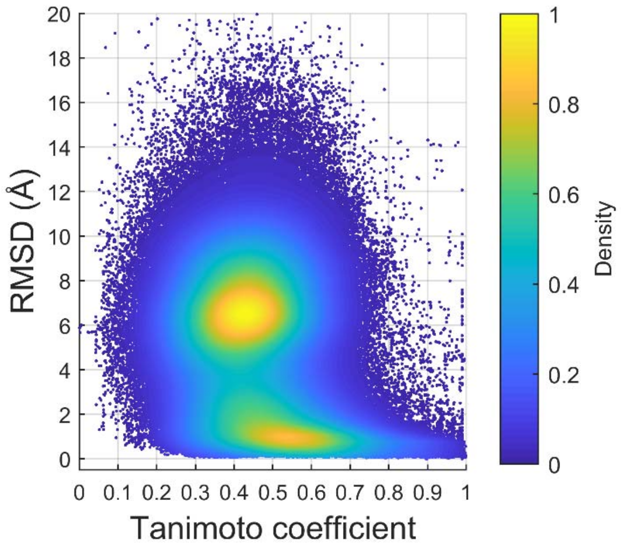

Figure S3 Elaborative studies of Figure 2(b). Distributions of the ligand RMSDs *vs* corresponding Tanimoto coefficients for all the cases in the protein-ligand dataset.
